# A Microfluidic Spheroid Culture Device with a Concentration Gradient Generator for High-Throughput Screening of Drug Efficacy

**DOI:** 10.3390/molecules23123355

**Published:** 2018-12-18

**Authors:** Wanyoung Lim, Sungsu Park

**Affiliations:** 1 Department of Biomedical Engineering, Sungkyunkwan University, Suwon 16419, Korea; wanyoung22@gmail.com; 2 School of Mechanical Engineering, Sungkyunkwan University, Suwon 16419, Korea

**Keywords:** spheroids, concentration gradient generator, drug screening, high-throughput

## Abstract

Three-dimensional (3D) cell culture is considered more clinically relevant in mimicking the structural and physiological conditions of tumors in vivo compared to two-dimensional cell cultures. In recent years, high-throughput screening (HTS) in 3D cell arrays has been extensively used for drug discovery because of its usability and applicability. Herein, we developed a microfluidic spheroid culture device (μFSCD) with a concentration gradient generator (CGG) that enabled cells to form spheroids and grow in the presence of cancer drug gradients. The device is composed of concave microwells with several serpentine micro-channels which generate a concentration gradient. Once the colon cancer cells (HCT116) formed a single spheroid (approximately 120 μm in diameter) in each microwell, spheroids were perfused in the presence of the cancer drug gradient irinotecan for three days. The number of spheroids, roundness, and cell viability, were inversely proportional to the drug concentration. These results suggest that the μFSCD with a CGG has the potential to become an HTS platform for screening the efficacy of cancer drugs.

## 1. Introduction

In general, two-dimensional (2D) cell culture models have been used for evaluating toxicity or effectiveness of drug candidates [1]. However, it has been shown that (a) 2D cell culture models are relatively poor in predicting drug responses, and (b) there are differences in the functional and phenotypic features between 2D and 3D cell cultures [2,3]. 3D cell culture models represent the in vivo microenvironments more accurately, and their predictability of drug effectiveness is better [4,5,6]. Recently, high-throughput screening (HTS) techniques of drugs have incorporated 3D cell cultures, and have rapidly progressed in the testing and selection of these drugs [7,8,9]. Accordingly, 3D cell arrays are extensively used nowadays for drug screening applications. From these, scaffold-free, 3D cell arrays—such as low-adhesion plates, micro-patterned plates, and hanging drop micro-plates—are the most commonly used at the present time [10,11,12,13]. The self-aggregation of cells is the key point of these methods. Moreover, these 3D cell arrays would include scaffolds, such as hydrogels and meshes, to mimic cell-to-extracellular matrix interactions and tissue-specific properties [14,15]. These micro-plate-based 3D cell arrays need complicated equipment such as robot arm, detectors and software for handling solution and data processing [16]. Furthermore, they are associated with several advantages, such as their reproducibility, simplicity of use for handling cultures, and abilities to treat and routinely analyse multi-cellular spheroids. However, this robotic equipment still suffers from several technical issues, such as high costs, poor handling of small volumes of liquid, and cell culture contamination [17]. The cost of devices and reagents for drug screening is also high because of high-volume consumptions. Therefore, new HTS systems combined with microfluidics, which require low sample and liquid volumes, are affordable, can easily handle small liquid volumes, enable serial processing and analysis, and are in the immediate needs for the drug development industry.

Microfluidic spheroid formation platforms have been applied to HTS for long-term perfusion cell cultures and have maintained high-cell viability. In the past, numerous microfluidic systems have been developed for formation of spheroids using microwells or U-shaped microstructures in the device. Microwell-based microfluidic platforms have been utilised more than other methods owing to their simplicity and easy operation [18,19,20]. Liu et al. designed a microfluidic device with U-shaped microstructures for spheroid formation and HTS [21]. These platforms were often combined with a concentration gradient generator (CGG) as a mixing channel [22,23,24]. Such a channel can be controlled for precise flow control. Recently, Fan et al. reported a high-throughput drug screening brain cancer chip composed of a photo-polymerised hydrogel to form multiple cancer spheroids [25]. They demonstrated that the culture array in association with a gradient generator was capable of forming spheroids, and for widespread parallel testing of drug responses. However, their microfluidic chip is difficult to be commercialised because of the short storage time of the hydrogel. In addition, because cells are injected through inlets, it is difficult for cells to go into the microwells through sub-channels, and their losses are thus high.

In this study, we developed a microfluidic spheroid culture device (μFSCD) with a CGG for evaluating the efficacy of cancer drugs to spheroids. In the device made of polydimethyl siloxane (PDMS), colon cancer cells (HCT116) were first deposited in concave microwells and later adhered to each other because they were not able to attach to the surface that was coated with bovine serum albumin (BSA). Once spheroids formed in this device, they were perfused with the anti-neoplastic enzyme inhibitor irinotecan (Camptosar^®^) to demonstrate the feasibility of the device for HTS applications. The device allowed cells to grow in 3D and to be perfused at different drug concentrations.

## 2. Results

### 2.1. Design of the μFSCD with a CGG

**Figure 1:**
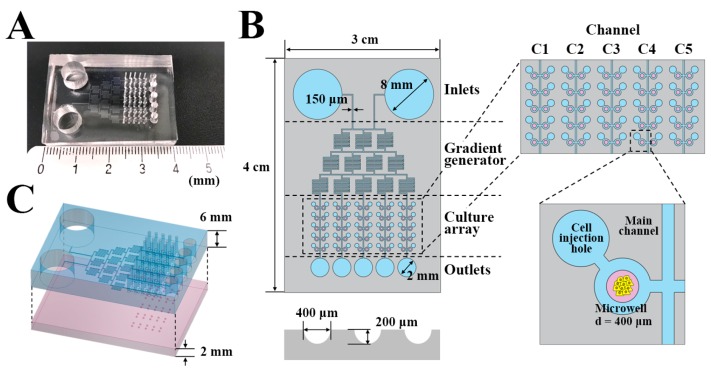
Design of the μFSCD with a concentration gradient generator (CGG). View (**A**) and dimensions (**B**) of the μFSCD with a CGG; (**C**) schematic showing two layers in the μFSCD with a CGG.

The dimensions of μFSCD with a CGG were 4 cm (L) × 3 cm (W) × 0.8 cm (H) (Figure 1). The device consists of two layers. The top layer has a 6 mm thickness and contains two inlets (8 mm in diameter), a gradient generator with 150 μm micro-channels, a culture array with fifty cell injection holes (700 μm diameters) and five outlets (2 mm diameters). The bottom layer is 2 mm in thickness and contains 50 concave microwells (400 μm in diameter and 200 μm in depth), and each channel (C1–C5) has 10 concave microwells (Figure 1B,C).

### 2.2. Concentration Gradient on the μFSCD with a CGG

**Figure 2:**
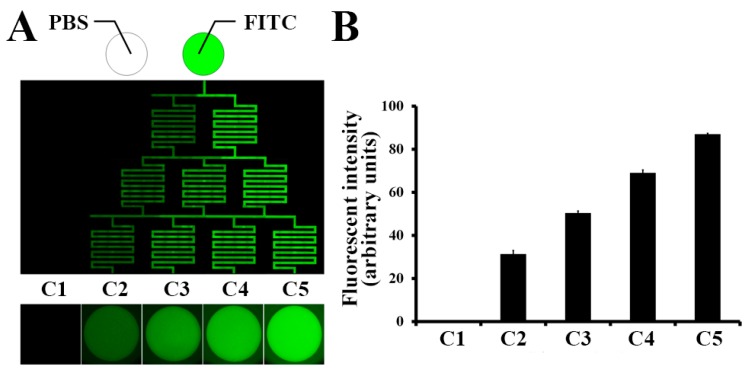
Concentration gradient of fluorescein isothiocyanate (FITC) in the μFSCD with a CGG. (**A**) Fluorescent image of FITC in the channels and concave microwells (C1–C5) 16 h after injection of phosphate-buffered saline (PBS) and FITC into the left and right inlets; (**B**) Fluorescent intensities of the microwells (C1–C5). *n* = 10.

To quantify the concentration gradient, the same volume (250 μL) of phosphate-buffered saline (PBS) and 5 μM fluorescein isothiocyanate (FITC) in PBS were filled into the left and right inlets. Fluorescent images were taken every 8 h and the concentration gradient was maintained for 16 h. Different intensities of fluorescence were observed through the parallel channels (Figure 2A). The FITC intensity of C5 was about 85% of the intensity of 5 μM FITC and highest among the microwells (C1–C5). The intensities of the wells decreased gradually from C5 to C1 (Figure 2B).

### 2.3. Spheroid Formation in the μFSCD with a CGG

**Figure 3:**
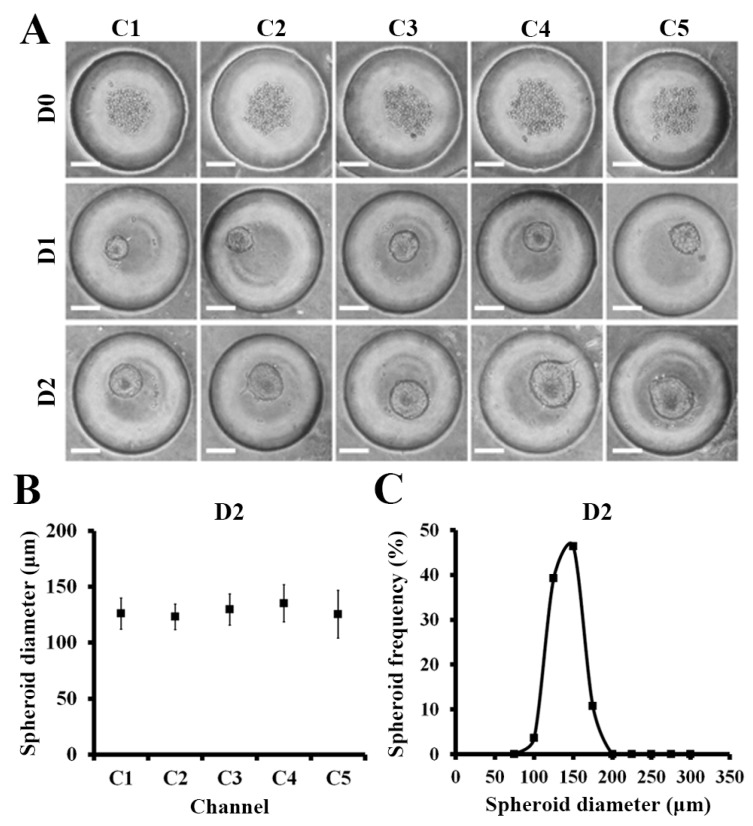
HCT116 spheroid formation in the μFSCD with a CGG at different days (0–2). (**A**) Optical images of spheroids formed in concave microwells. Scale bars, 100 μm; (**B**) Spheroid diameters in each channel at D2 (*n* = 10); (**C**) Spheroid diameter frequency distribution at D2 (*n* = 50).

Approximately 200 cells were injected through the cell injection holes and approximately 110 cells per microwell were captured. The capturing efficiency was approximately 50%. The captured cells were then cultured in the device. They formed spheroids at D1 and became larger at D2 (Figure 3A). The average diameter of the spheroids (*n* = 50) formed in microwells at D2 was 128.1 ± 16.6 μm, thereby indicating that the spheroids were homogeneous (Figure 3B). The distribution of the fifty spheroids as a function of their diameters was also uniform (Figure 3C). Similarly, glioma cell line (U87) were formed homogeneous spheroids and the average diameter of them at D2 was 204.2 ± 9.7 μm (see Supplementary Figure S2).

### 2.4. Drug Screening for Spheroids in the μFSCD with a CGG

**Figure 4:**
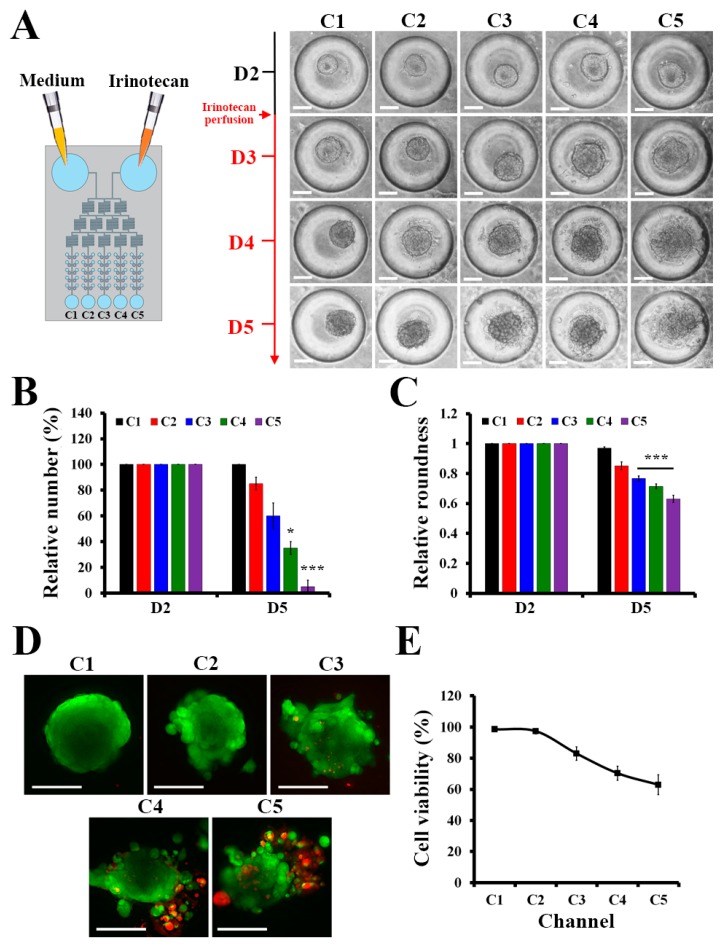
Responses of HCT116 spheroids to irinotecan in the μFSCD with a CGG. (**A**) Optical images of HCT116 spheroids with the treatment of 5 μM irinotecan at various days (D2–D5) (Scale bars, 100 μm). Relative numbers (**B**) and relative roundness values (**C**) of HCT116 spheroids in each channel at D2 and D5 (*n* = 20, Student’s *t*-test, * *p* < 0.05, *** *p* < 0.001). Live/dead staining images (**D**) and cell viability (**E**) of HCT116 spheroids treated with irinotecan in each channel at D5 (Scale bars, 100 μm). Calcein AM and EthD-1 were used to stain live and dead cells in green and red, respectively.

Once spheroids formed, 5 μM irinotecan which is 8 times higher half maximal inhibitory concentration in 2D culture of HCT116 (Supplementary Figure S1) was injected into the device to generate the concentration gradient of the drug, and the responses of the spheroids in the presence of the gradient were investigated. Figure 4A showed that the spheroids in C1 became larger, while the structures of the spheroids from C2 to C5 became loose, and cells detached from the spheroids. The relative number of HCT116 spheroids in C4 and C5 at D5 was significantly lower than those of the HCT116 spheroids in C1, C2, and C3 (Figure 4B). The roundness of spheroids [26] can be used to determine whether the spheroids were affected by the drug. HCT116 spheroids treated with irinotecan were collapsed. Thus, the roundness of the spheroids in C3, C4, and C5 decreased over time, whereas the roundness of the spheroids in C1 and C2 remained similar (Figure 4C). This indicates that the CGG structure was appropriate to generate the expected drug concentration gradient. These results were supported by live/dead staining images in Figure 4D. Accordingly, C5 yielded the most collapsed spheroid, while the dead cells, which are stained in red, indicated that the concentration of irinotecan in C5 was higher than the concentrations of the other channels. The cell viability was measured by live/dead staining in Figure 4E. At D5, after irinotecan treatment, the cell viability of C5 was the lowest with approximately 63%, while the cell viability in C1 was 98%. The cell viabilities reduced continually following the drug concentration in each channel. These results showed the potential application of the μFSCD with a CGG in HTS.

## 3. Discussion and Conclusions

In this study, we developed a microfluidic spheroid culture device with a CGG for 3D spheroid cultures for high-throughput drug screening. Common microfluidic systems are complicated and use a syringe pump to perfuse nutrients, drugs, and cells [27,28]. Our μFSCD with a CGG has reservoirs instead of a syringe pump, and could perfuse medium into spheroids in concave microwells. In the μFSCD with the CGG, HCT116 cells were deposited in concave microwells and were not attached to the PDMS surface because of the BSA coating, and concave shape. Concave shape microwell makes cells aggregate and form spheroid due to the low cell attachment to surfaces [29]. Generally, large spheroids without vascularisation can induce cell death because of oxygen deficiency at the center. Thus, it is important to produce relatively small spheroids. The thickness of the spheroids cultured under optimal nutrient and oxygen conditions ranged from 100 to 220 μm [30], so the spheroids with diameters in the range of 100 to 150 μm were selected for drug responses. As a result of the optimisation of the cell density to 5 × 10^4^ cells/mL, relatively similar size spheroids with an average diameter of 120 μm were obtained in the μFSCD with a CGG (Figure 2). Although our experiments were performed with the use of a spheroid with an average diameter of 120 µm in size, the size of the spheroid can be controlled based on the cell density [25]. The uniformness of spheroids is also critical to get precise drug responses. When the spheroids size is not uniform, the permeability of nutrients, gas, and drug is different in each spheroid, and elicits diverse drug responses [31,32,33]. The μFSCD with a CGG can generate homogeneous spheroids because it is easy to load a similar number of cells through the cell injection hole.

Gradient generation by CGG provided five different irinotecan concentration conditions and was suited for HTS with a large number of spheroids in parallel. Each generated concentration from CGG can be calculated easily. When only two inlets are included, intermediate values between two neighboring concentrations are generated regardless of what the splitting ratios are and thus the gradient profile is always kept monotonous [34,35]. Thus, the concentration of treated irinotecan was expected to 0, 1.25, 2.5, 3.75 and 5 μM (C1–C5). CGG has been included in many microfluidic systems for drug screening because it generates various drug concentrations by perfusing a single concentration of the drug. Additionally, its concentration is predictable so it is advantageous to identify the appropriate concentration of the drug [35]. Furthermore, PDMS are proper for extending the duration of the chemical gradient because of their evaporation and liquid absorption functions [36]. In the future, the number of concentration points can be increased to handle more drug concentrations at any time instant.

In addition, the numbers and shapes of spheroids can be easily observed under an optical microscope in label-free conditions owing to the transparent PDMS substrate (Figure 3). Spheroid disaggregation increased after drug treatment. Thus, it was difficult to compare the drug effects by measuring the spheroid diameters. Instead of measuring the diameter, the surface of the spheroids was measured [37], and spheroids which displayed a rough surface were not counted (Figure 3B). The roundness of the spheroids elicited more accurate drug response results as their spheroid shapes were collapsed by the drug (Figure 3C). Furthermore, viability analysis by live/dead staining can provide a sensitive measure of spheroid responses to a particular drug (Figure 4). Spheroids can be easily stained through the cell injection holes.

In conclusion, our μFSCD with a CGG offered a new approach for large-scale drug screening using spheroid microarrays. This approach was based on features of concave microwells connected with a CGG. Given that the μFSCD with the CGG provided a large amount of homogeneous 3D spheroids and several drug concentrations, it can constitute a convenient tool for widespread, parallel processing for the prediction of the effectiveness of the drugs and the determination of the proper cancer drug concentration in patients. Therefore, the proposed μFSCD with a CGG could be useful for clinical samples and could become cost-effective in personalised medicine given that a small number of cells can rapidly form spheroids, and given that the device consumes a small volume of nutrients and drugs.

## 4. Materials and Methods

### 4.1. Design and Fabrication of the Device

The μFSCD with a CGG was designed with AutoCAD (Student version, Autodesk Korea Limited, Seoul, Korea, 2015). The design was printed in a transparent film. For fabricating a mold with the design, the negative photoresist SU–8 (MicroChem Corp., Westborough, MA, USA) was spin-coated on a 4 in silicon wafer for 30 s (top layer–SU–8 2025, 2000 rpm, 40 μm thickness; bottom layer–SU–8 2150, 2000 rpm, 300 μm thickness). The coated wafer was exposed to UV through the film using a contact aligner (MDA–400M, MIDAS SYSTEM CO., Daejeon, Korea). The exposed wafer was developed with an SU–8 developer (MicroChem Corp.) to obtain the mold. Then, the mold was treated with trichloro (1H,1H,2H,2H-perfluorooctyl) silane (Sigma–Aldrich, St. Louis, MO, USA) in vacuum conditions at RT for 1 h. Both layers were made from PDMS (Sylgard^®^ 184, Dow Corning Corp., Midland, MI, USA) using soft lithography [38]. Fifty cell injection holes (700 μm diameter), two inlets (8 mm diameter), and five outlets (2 mm diameter) were punched through the top layer. For the manufacturing of the bottom layer with the concave microwells, the cylindrical microwells were filled with PDMS and removed by wiping. Remained PDMS in the cylindrical microwells formed a concave meniscus. Details can be found in a previous publication [39]. Subsequently, both layers were treated with O_2_ plasma for 30 s at 90 W, and were then bound to each other.

### 4.2. Demonstration of Concentration Gradients in the μFSCD with a CGG

The device was treated with 3% (*w*/*v*) BSA (Sigma–Aldrich) for 1 h at RT and then rinsed three times with PBS (pH = 7.4). The inlets were filled with 250 μL of PBS, and PBS contained 5 μM FITC Fluorescent images were acquired every 8 h using a DeltaVision Elite fluorescence microscope (GE Healthcare, Chicago, IL, USA). Fluorescent intensity profiles over the μFSCD with a CGG were analysed with ImageJ (Fiji, NIH, Bethesda, MD, USA).

### 4.3. Cells and Their Maintenance

The colon cancer cell line (HCT116) and glioma cell line (U87) was purchased from ATCC (Manassas, VA, USA). HCT116 and U87 was cultured in McCoy’s 5A Medium and Minimum Essential Media (Life Technologies, Carlsbad, CA, USA) supplemented with 10% (*v*/*v*) fetal bovine serum (HyClone Laboratories, Inc., Logan, UT, USA), 100 units per mL of penicillin (Life Technologies) and 100 μg/mL of streptomycin (Life Technologies). The cells were maintained at 37 °C with 5% CO_2_.

### 4.4. Operation of the μFSCD with a CGG

For sanitisation, the μFSCD with a CGG was washed with 70% ethanol through one of the reservoirs, and was rinsed with PBS using a 1 mL syringe. The μFSCD was treated with 3% BSA for 1 h at RT to avoid cell attachment to the bottom. It was rinsed again two times with PBS to remove the uncoated BSA from it. The sanitised device was washed with medium through one of the reservoirs using a 1 mL syringe, and the inlets were filled with 250 μL of medium. Approximately 1 μL of 2 × 10^4^ cells/mL in medium were loaded into the device through each cell injection hole using a pipette. Uncaptured cells in the device were removed by pipetting. The device, which contained the cells, was incubated at 37 °C in a 5% CO_2_ incubator for 2 days to generate spheroids. After 2 days, inlets were filled with 250 μL of medium, and contained 5 μM irinotecan and medium. The inlet contents were replaced daily with fresh medium and contained 5 μM irinotecan or medium, and the outlet contents were removed for 3 days.

### 4.5. Live/Dead Staining and Cell Viability Measurements

HCT116 spheroids at D5 after irinotecan treatment were treated with a LIVE/DEAD™ Viability/Cytotoxicity Kit (Molecular Probes, Eugene, OR, USA), as suggested by the manufacturer. The inlets were filled with the mixed live/dead solution and then incubated at 37 °C for 30 min. Green and red fluorescence intensities were measured with ImageJ, and cell viability was calculated as the ratio of the red with the green fluorescence intensities.

## Supplementary Materials

The following are available online, Figure S1: Relative cell viability of HCT116 monolayers with the treatment of irinotecan at different concentrations (0–100 μM) for 72 h. Cell viability was measured using the EZ-cytox Cell Viability Assay Kit (Daeillab Service, Seoul, Korea), Figure S2: U87 spheroid formation in the μFSCD with a CGG at different days (0–2). (A) Optical images of spheroids formed in concave microwells. Scale bars, 100 μm; (B) Spheroid diameters in each channel at D2 (*n* = 10); (C) Spheroid diameter frequency distribution at D2 (*n* = 50).
