# Supplementary material for: A Microfluidic Spheroid Culture Device with a Concentration Gradient Generator for High-Throughput Screening of Drug Efficacy
Source: Molecules. 2018 Dec 18;23(12):3355. doi: 10.3390/molecules23123355 (PMC6321514; doi:10.3390/molecules23123355)
Supplement: Supplementary file 1 [file molecules-23-03355-s001.pdf]

Supplementrary Materials to A microfluidic spheroid culture device with a concentration gradient generator for high-throughput screening of drug efficacy

Wanyoung Lim[1] and Sungsu Park[1,2,*]

[1] Department of Biomedical Engineering, Sungkyunkwan University, Suwon, Korea.
[2] School of Mechanical Engineering, Sungkyunkwan University, Suwon, Korea.
[*] Corresponding author: S. Park. Address: School of Mechanical Engineering, Sungkyunkwan University, Seobu-ro, Suwon 16419, Kyunggi-do, Republic of Korea/Email: nanopark@skku.edu/ Tel: +82-31-290-7431/Fax:+82-31-290-5889

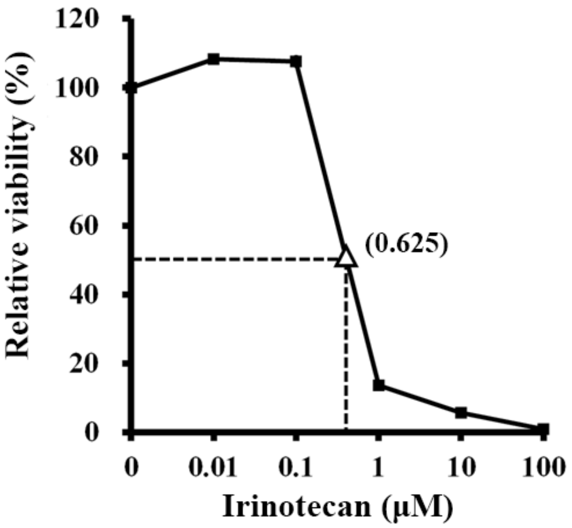

Figure S1. Relative cell viability of HCT116 monolayers with the treatment of irinotecan at different concentrations (0–100 μM) for 72 h. Cell viability was measured using the EZ-cytox Cell Viability Assay Kit (Daeillab Service, Seoul, Korea).

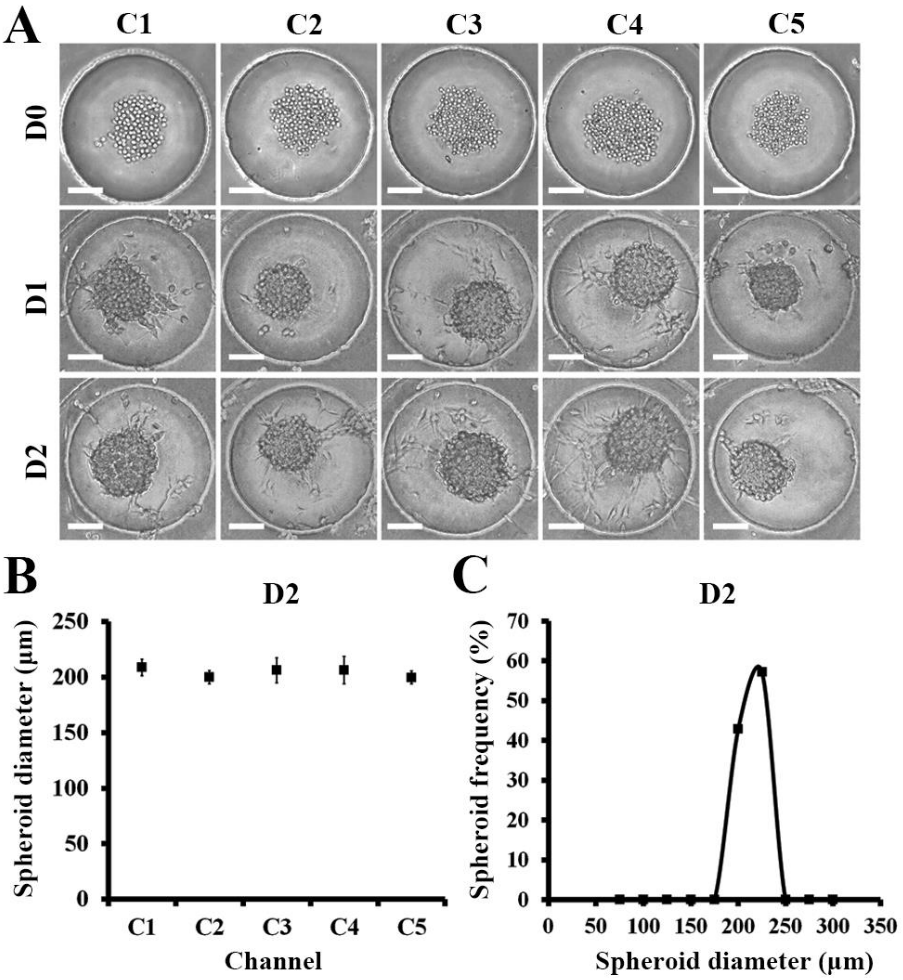

Figure S2. U87 spheroid formation in the µFSCD with a CGG at different days (0–2). (A) Optical images of spheroids formed in concave microwells. Scale bars, 100 µm; (B) Spheroid diameters in each channel at D2 (n = 10); (C) Spheroid diameter frequency distribution at day 2 (n = 50).
